# Mapping neuroinflammation in Parkinson's brain with novel imaging biomarkers

**DOI:** 10.1002/alz70855_105602

**Published:** 2025-12-24

**Authors:** Avishek Roy, Filipa Monteiro Rocha, Mukesh Varshney, Amit Kumar

**Affiliations:** ^1^ Karolinska Institutet, Stockholm, Sweden

## Abstract

**Background:**

Despite significant glial involvement in Parkinson's disease (PD), the knowledge regarding the neuroinflammatory role of sustained glial cells (astrocyte and microglia) activation is still at the surface level; largely due to a lack of specific biomarkers to track these processes. Novel astroglial (BU99008 and Deprenyl) and microglial (ER176 and PK11195) positron emission tomography (PET)‐tracers hold immense potential for visualizing reactive astrocytes and activated microglia in PD. However, they have not been thoroughly investigated in PD.

**Method:**

Hence, we employed a multi‐PET tracer approach and performed specialized radioligand binding assays and postmortem brain imaging autoradiography studies, complemented with astrocytes/microglial morphometric and markers (GFAP, S100B, and IBA1) analyses to elucidate the contribution of glial‐mediated inflammation in PD brains.

**Result:**

Radioligand binding studies demonstrated distinct tracer binding behavior with 3H‐BU99008 and 3H‐Deprenyl showing diverse (single or multiple) binding sites in a region‐dependent manner. Autoradiography studies complemented these observations and showed that 3H‐BU99008 and 3H‐Deprenyl might be targeting different subpopulations astrocytes in CN and PD brains. Importantly, we observed **significant reactive astrogliosis in the PD brain regions as compared to CNs** marked by prominent changes in astrocytic morphology and cellular processes, and upregulation of a specific astrocytic subpopulation; GFAP/ICAM1 positive‐reactive astrocytes. ICAM1 is a cell‐surface glycoprotein heavily involved in immune/cellular inflammatory responses and a ligand for microglial‐receptors.

**Conclusion:**

This study is the first to provide a complete snapshot of reactive astrogliosis at the advanced/end stages of PD and demonstrate a much‐pronounced role of astrocytic ICAM1 in PD pathogenesis. Our ongoing mechanistic and translational studies with microglial tracers/markers will shed more light on the interplay between astrocyte‐microglia activation in PD pathogenesis and further elucidate the unexplored intermediary role of ICAM1 in PD astrocyte reactivity and subsequent microglial neuroinflammatory response.

